# Validation of the BioIntelliSense BioButton® device for physical activity monitoring in children and future application as a physical health outcome for critically Ill children

**DOI:** 10.3389/fped.2025.1544404

**Published:** 2025-04-15

**Authors:** Lexi Petruccelli, Kristen R. Miller, Rachel Greer, Heidi Sauceda, R. Scott Watson, Peter M. Mourani, Aline B. Maddux

**Affiliations:** ^1^ Research Institute, Pediatric Critical Care, Children’s Hospital Colorado, Aurora, CO, United States; ^2^ Department of Pediatrics, University of Colorado School of Medicine, Aurora, CO, United States; ^3^ Department of Pediatrics, Division of Critical Care, University of Washington, Seattle, WA, United States; ^4^ Seattle Children’s Research Institute, Seattle Children’s Hospital, Seattle, WA, United States; ^5^ Department of Pediatrics, Section of Critical Care, University of Arkansas for Medical Sciences and Arkansas Children’s, Little Rock, AR, United States; ^6^ Department of Pediatrics, Section of Critical Care Medicine, University of Colorado School of Medicine, Aurora, CO, United States; ^7^ Children’s Hospital Colorado, Aurora, CO, United States

**Keywords:** exercise, sedentary behavior, patient outcome assessment (MeSH), pediatrics—children, critical care outcomes

## Abstract

**Introduction:**

Mobile monitoring devices offer an opportunity to characterize physical health recovery in children who survive critical illness.

**Methods:**

To validate the BioIntelliSense BioButton® as a pediatric activity monitor, we studied healthy children (2–17 years-old) who wore the BioButton® device and an ActiGraph wGT3X-BT accelerometer, and a study team member documented activity in 1 min intervals (gold standard) during 45 min of scripted activities. In two-thirds of the cohort (derivation cohort), we identified BioButton activity count thresholds to differentiate activity levels based on highest Youden indices. Thresholds were applied to the remainder of the cohort (validation cohort) to determine sensitivity and specificity [95% confidence interval (CI)]. We also evaluated BioButton activity designations compared with accelerometer designations and calculated agreement between BioButton-measured body position and the activity log.

**Results:**

Forty-five participants provided a median 43 (IQR 41, 44) analyzable minutes. Sensitivity and specificity of derived BioButton thresholds were 0.78 (95% CI: 0.69, 0.88) and 0.95 (95% CI: 0.90, 0.97) to identify moderate or vigorous activity (MVPA) and 0.91 (95% CI: 0.87, 0.95) and 0.98 (95% CI: 0.98, 0.98) to identify sedentary behavior. Sensitivity and specificity compared with the accelerometer were 0.52 (95% CI: 0.45–0.60) and 0.88 (95% CI: (95% CI: 0.84, 0.93) to identify MVPA and 0.92 (95% CI: 0.89–0.96) and 0.70 (95% CI: 0.67, 0.73) to identify sedentary behavior. The BioButton accurately identified position during 1,125 of 1,432 (78.6%) minutes.

**Discussion:**

The BioButton device accurately identified physical activity and body position in children and may be a useful tool to quantify physical activity as an outcome in future trials.

## Introduction

In 2019, an estimated 239,000 U.S. children were admitted to an intensive care unit (1). Although more than 97% of children survived, there is growing recognition that survivors of critical illness can suffer from long-lasting impairments associated with their critical illness (2). Notably, critical illness puts children at risk of prolonged impairments in physical functioning. In a prospective study of 144 children who were mechanically ventilated for at least 3 days, one in five children had persistently worse physical health-related quality of life (HRQL) relative to their pre-admission state that persisted for at least one year after discharge (3). Other studies demonstrated similar or higher rates of physical impairments after pediatric critical illness (4–6). Impairments in physical activity are critically important because prolonged impairment in physical functioning may limit a child's ability to participate in activities that foster physical, social, and cognitive development, decreasing the likelihood of maintaining a healthy lifestyle in adulthood.

As the focus on survivorship has increased, core outcome sets were developed to guide evaluation of critically ill children. Across all outcome sets, physical activity is identified as a critically important outcome (7–9). The measures used to evaluate physical activity are primarily survey-based and, while patient- and proxy-reported outcomes such as HRQL offer valuable insight into a patient's or caregiver's perception of their or their child's health, direct measurements offer complimentary information, providing a more comprehensive understanding of a patient's recovery (10–12). Additionally, in contrast to direct measurements of functional abilities that can be measured in the clinic setting (e.g., six-minute walk test), functional outcomes such as physical activity are best evaluated in a child's natural setting to incorporate the context of everyday living.

The rapid development of mobile technologies provides an opportunity to augment measurement of physical health outcomes in children (13–15). Activity monitors have been used in other domains of pediatric research to evaluate physical health (16–18), but their use to measure physical recovery after a critical illness is limited (19). To ensure feasibility for use in studies evaluating children who survive a critical illness, the activity monitor must be acceptable to wear, and participation should be facilitated remotely given the challenges of in-person contact with study participants after discharge. We previously used the ActiGraph accelerometer (Pensacola, Florida USA), worn on hip (<6 years old) and wrist (≥6 years old), to evaluate physical activity in children who survived an episode of acute respiratory failure (19). The study's impact was limited by missing data because of reluctance to wear the monitor due to discomfort and visibility. In this study, we assessed the BioIntelliSense BioButton® (Golden, Colorado USA) as an alternative device to measure activity in children. The BioButton® is a 1.5 inch-diameter device that is worn directly on the skin and uses accelerometer technology to measure activity. The BioButton received 510(k) clearance by the U.S. Food and Drug Administration (FDA) in December of 2022 and is available for use in pediatric research as a non-significant risk device (20). It has been used to monitor adults during and after hospitalization but has not been used clinically in children (21). The primary objective of this study was to evaluate the accuracy of the BioIntelliSense BioButton® device to characterize physical activity in children relative to direct observation and, as a secondary objective, we compared physical activity level as characterized by the BioButton to the ActiGraph wGT3X-BT accelerometer. We hypothesized that the BioIntelliSense BioButton® device accurately characterizes moderate or vigorous physical activity (MVPA) and sedentary behavior.

## Materials and methods

This study was approved by the Colorado Multiple Institutional Review Board (Protocol: #22-2214, Physical Activity Monitor Validation) on February 06, 2023, and the study was conducted in accordance with institutional standards and the 1975 Helsinki Declaration. Consent was obtained from each participant's legal guardian, and child assent was obtained in children 7 years and older. Participants were healthy volunteers recruited through advertisements in Children's Hospital Colorado research newsletters. We included children (age 2 to 17) who were ambulatory (defined as able to walk and run independently) and able to engage in age-appropriate activities. We excluded children with chest burns or wounds that would preclude placement of the BioButton device, those who were in state custody, or if they resided in juvenile detention or jail. Enrollment was stratified by age group [>2 to <6 years-old [early childhood], ≥6 to <12 years-old [middle childhood], and ≥12 to <17 years-old [adolescence]]. We enrolled healthy participants to facilitate the ability to directly monitor activity in a prescribed setting which would not have been feasible if we enrolled children who had survived critical illness and, although the amount of activity may differ, we anticipated activity monitors would perform similarly in healthy children and those surviving critical illness. We evaluated for eligibility using a pre-eligibility survey administered by telephone to the participants' parent or legal guardian.

Study participants engaged in directed activities including sedentary behavior and light, moderate, or vigorous activity for a total duration of approximately 45 min (Supplementary eTable S1) (22). During the activities, each participant wore the BioIntelliSense BioButton device, adhered to the left side of their chest, and the ActiGraph accelerometer on their waist (<6-years-old) or wrist (≥6-years-old) (23, 24). In older children, wrist-worn accelerometers provide comparable data and increase wear time as compared to hip accelerometers (25–28). In younger children, hip worn accelerometers are shown to be well tolerated (29). We used previously reported thresholds to characterize activity as sedentary behavior or light, moderate, or vigorous activity based on vector magnitude data reported by the ActiGraph wGT3X-BT accelerometer (23, 24). The BioButton and ActiGraph accelerometer use triaxial accelerometer technology to measure activity defined as activity counts for the BioButton and vector magnitudes for the ActiGraph accelerometer. The BioButton also detects body position and classifies it as supine, prone, lateral, or upright (sitting or standing). A study team member assigned to each participant documented activity, activity level, and position in 1 min intervals in the participant's activity log. At the end of the 45 min activity period, surveys were distributed to families to evaluate receptivity of participants in using the devices (Supplementary eTable S2). The study was conducted at a local park over the course of five days during the summer months under similar weather conditions.

### Statistical analysis

Variables were summarized as median and interquartile range (IQR) or frequency and percentage. To determine BioButton thresholds to characterize activity level in children, the cohort was divided into derivation and validation cohorts. Data from the derivation cohort identified optimal BioButton activity count thresholds to differentiate between the four different levels of activity (sedentary, light, moderate, and vigorous) as compared with the activity log, considered to be the gold standard. Receiver operating characteristic (ROC) curves were created for each pairwise activity level, as recorded by the activity log: (1) sedentary vs. light, (2) light vs. moderate, (3) moderate vs. vigorous. The optimal cutpoint between each level was chosen as the activity count with the highest Youden index (sensitivity + specificity −1) to optimize sensitivity and specificity. We calculated sensitivity and specificity of the thresholds to identify MVPA and sedentary behavior across the derivation cohort and validation cohort, using the activity log as the gold standard. This was repeated within each age group. Due to the clustered nature of the repeated measures on a single child, the ratio estimator for the variance of clustered binary data was used to calculate the confidence intervals of sensitivity and specificity (30, 31). As a secondary comparison, we evaluated the BioButton-derived activity level with the activity level characterized by the ActiGraph accelerometer in the full study cohort. To evaluate accuracy of the BioButton device to characterize body position, data from all participants were used to assess the percent agreement between body position designations by the BioButton device and the activity log. Sensitivity and specificity for correctly identifying body position were also reported. Missing data were not imputed. Statistical analyses were conducting using R version 4.2.2 (Vienna, Austria).

## Results

We enrolled 45 children, 28 in the derivation cohort and 17 in the validation cohort. Participants were divided into the validation and derivation cohorts based on timing of participation in the study. Patient characteristics were similar between the derivation and validation cohorts, and there were at least 5 participants evaluated in each age group in both the derivation and validation cohorts (Table 1). Study activities were conducted outdoors over 5 days between July and September of 2023. On the study days, daily high temperatures ranged from 80 to 88 degrees Fahrenheit. Participants engaged in study activities for a median of 46 min (IQR 45, 49) and participants had a median of 43 (IQR 41, 44) analyzable minutes with BioButton, ActiGraph accelerometer, and activity log information.

**Table 1:** Study cohort.

Characteristics	Total (*n* = 45)	Derivation (*n* = 28)	Validation (*n* = 17)
Age group, *n* (%)			
>2 and <6 years	15 (33.3)	9 (32.1)	6 (35.3)
≥6 and <12 years	19 (42.2)	14 (50.0)	5 (29.4)
≥12 and <17 years	11 (24.4)	5 (17.9)	6 (35.3)
Female sex, *n* (%)	26 (57.8)	15 (53.6)	11 (64.7)
BMI, median (IQR)	16.7 (14.7, 18.3)	16.0 (14.8, 17.5)	17.6 (14.6, 19.7)

IQR, interquartile range.

We used data from the derivation cohort to identify optimal activity count thresholds measured by the BioButton to identify sedentary behavior and light, moderate, and vigorous activity based on activity log data (Supplementary eTable S3). In the derivation subset of the cohort, sensitivity for identifying MVPA was 0.76 (95% CI: 0.69, 0.82) and specificity was 0.91 (95% CI: 0.89, 0.94) (Table 2). Across the three age groups, sensitivity values were greater than 0.75 with exception of the ≥12 year-old age group [0.63 (95% CI: 0.45, 0.80)], and specificities were greater than 0.9. Sensitivity of the BioButton device for identifying sedentary behavior was 0.88 (95% CI: 0.85, 0.91), and specificity was 0.93 (95% CI: 0.91, 0.95). Subsequently, we tested these thresholds in the validation cohort. Sensitivity for identifying MVPA was 0.78 (95% CI: 0.69, 0.88), and specificity was 0.95 (95% CI: 0.90, 0.97). Similar sensitivities and specificities were demonstrated across the three age groups (Table 3). Sensitivity of the BioButton for identifying sedentary behavior was 0.91 (95% CI: 0.87, 0.95) and specificity was 0.98 (95% CI: 0.98, 0.98) with similar performance across the three age groups (Table 2).

**Table 2:** Discrimination values of the bioButton compared with the activity Log: derivation cohort.

Cohort or cohort subset	Sensitivity (95% confidence interval)	Specificity (95% confidence interval)
Derivation cohort: discrimination of moderate or vigorous physical activity		
Entire cohort	0.76 (0.69, 0.82)	0.91 (0.89, 0.94)
>2 and <6 years	0.77 (0.65, 0.89)	0.90 (0.86, 0.94)
≥6 and <12 years	0.80 (0.72, 0.88)	0.91 (0.86, 0.95)
≥12 and <17 years	0.63 (0.45, 0.80)	0.95 (0.93, 0.97)
Derivation cohort: discrimination of sedentary behavior		
Entire cohort	0.88 (0.85, 0.91)	0.93 (0.91, 0.95)
>2 and <6 years	0.90 (0.85, 0.95)	0.94 (0.89, 0.98)
≥6 and <12 years	0.86 (0.81, 0.91)	0.94 (0.92, 0.96)
≥12 and <17 years	0.89 (0.88, 0.91)	0.89 (0.86, 0.92)
Validation cohort: discrimination of moderate or vigorous physical activity		
Entire cohort	0.78 (0.69, 0.88)	0.95 (0.90, 0.97)
>2 and <6 years	0.74 (0.59, 0.89)	0.94 (0.89, 0.98)
≥6 and <12 years	0.81 (0.65, 0.98)	0.92 (0.85, 0.99)
≥12 and <17 years	0.80 (0.62, 0.99)	0.98 (0.95, 1.00)
Validation cohort: discrimination of sedentary behavior		
Entire cohort	0.91 (0.87, 0.95)	0.98 (0.98, 0.98)
>2 and <6 years	0.89 (0.80, 0.98)	0.99 (0.99, 0.99)
≥6 and <12 years	0.89 (0.85, 0.93)	0.98 (0.95, 1.00)
≥12 and <17 years	0.95 (0.91, 1.00)	0.98 (0.96, 1.00)

**Table 3:** Discrimination values of the bioButton compared with the actiGraph accelerometer^a^.

Cohort or cohort subset	Sensitivity (95% confidence interval)	Specificity (95% confidence interval)
Discrimination of moderate or vigorous physical activity		
Entire cohort	0.53 (0.47, 0.58)	0.89 (0.85, 0.93)
>2 and <6 years	0.81 (0.72, 0.91)	0.82 (0.76, 0.88)
≥6 and <12 years	0.50 (0.44, 0.56)	0.98 (0.96, 1.00)
≥12 and <17 years	0.43 (0.34, 0.52)	1.00 (1.00, 1.00)
Discrimination of sedentary behavior		
Entire cohort	0.94 (0.91, 0.97)	0.71 (0.68, 0.73)
>2 and <6 years	0.92 (0.88, 0.96)	0.80 (0.76, 0.85)
≥6 and <12 years	0.93 (0.87, 0.98)	0.67 (0.64, 0.70)
≥12 and <17 years	1.00 (1.00, 1.00)	0.66 (0.63, 0.69)

^a^ Participants wore ActiGraph Accelerometer on the waist (<6-years-old) or wrist (≥6-years-old).

We compared the activity level identified by the BioButton with the activity level identified by the ActiGraph accelerometer as a secondary gold standard (Table 3). Compared with the ActiGraph accelerometer, the BioButton demonstrated a sensitivity of 0.52 (95% CI: 0.45, 0.60) and specificity of 0.88 (95% CI: 0.84, 0.93) in identifying MVPA. Sensitivities were lower in older age groups, but specificities were similar. Compared with the ActiGraph accelerometer, the BioButton demonstrated a sensitivity of 0.92 (95% CI: 0.89, 0.96) and specificity of 0.70 (95% CI: 0.67, 0.73) in identifying sedentary behavior. Performance was similar across the three age groups.

To evaluate accuracy of the BioButton to identify body position, we analyzed 1,432 min. Of these 1,432 min, the device accurately identified participant position during 1,125 (78.6%) minutes (Supplementary eTable S4). Of the 947 min of upright positioning (sitting and standing), the BioButton device accurately identified 861 (90.9%) minutes as upright (Supplementary eTable S4). Sensitivity for identifying upright position was 0.91 (95% CI: 0.88, 0.94) and specificity was 0.76 (95% CI: 0.69, 0.83) (Supplementary eTable S5). Of the 485 min of recumbent positioning, the device accurately identified 317 (72.9%). For the recumbent positions, sensitivity was 0.76 (95% CI: 0.69, 0.83) and specificity was 0.91 (95% CI: 0.88, 0.94). Notably, supine position was most often categorized as upright (Supplementary eTable S6).

**Figure 1:**
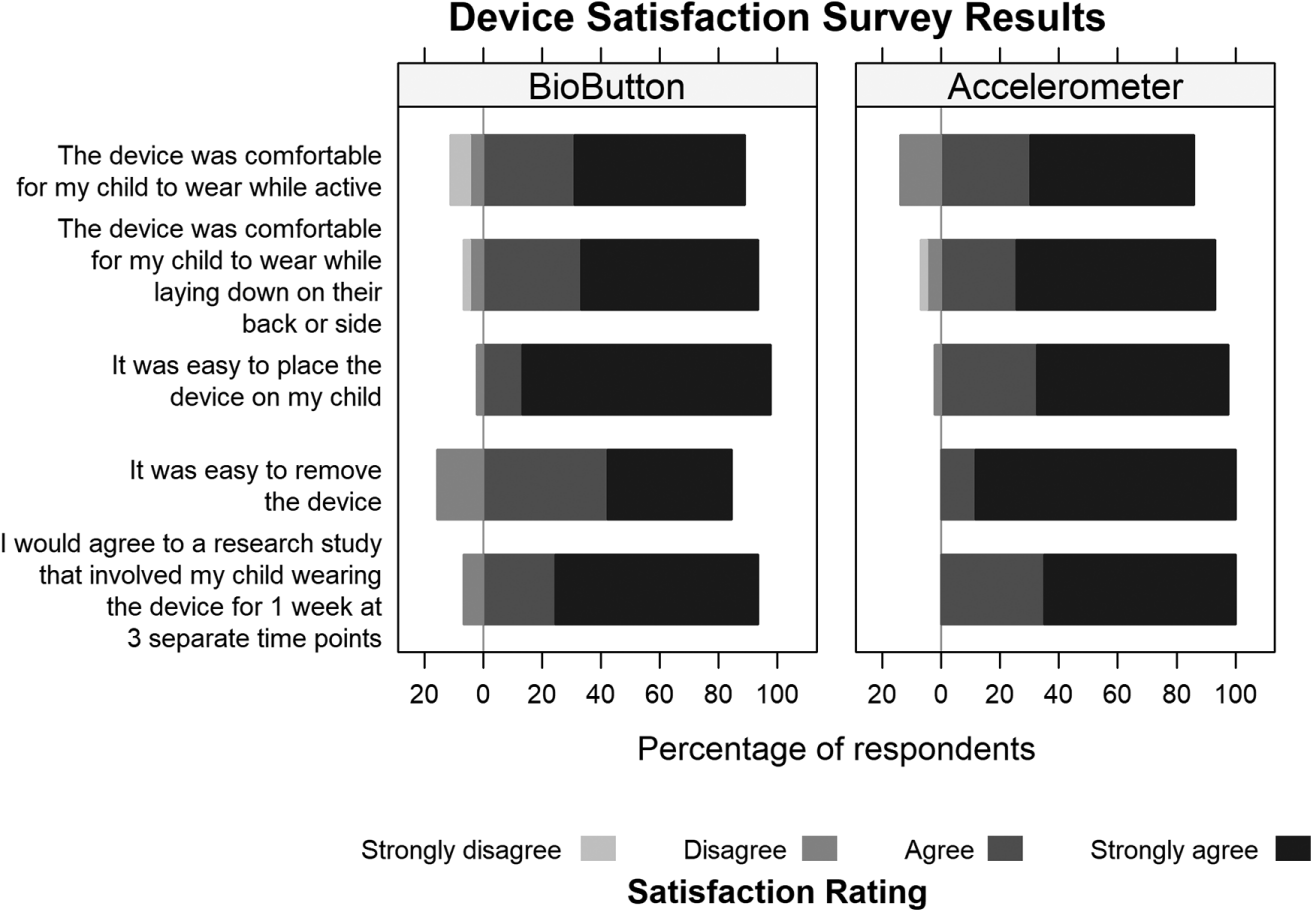
Satisfaction survey results.

Surveys completed at the end of the participation period demonstrated that nearly all participants agreed or strongly agreed that the devices were comfortable to wear and easy to place and remove, and they would agree to wear the devices for the conduct of a study (Figure 1). There was no difference in survey responses based on age <6 and ≥6 years-old (data not shown). As noted by two participants in our study, the BioButton device adhesive was less effective when placed on moist skin such as would occur if placement occurred during activity causing extreme perspiration. If the adhesive was placed prior to sweating, it was more effective.

## Discussion

In this cohort of healthy, typically developing children, we identified that the BioButton device was able to differentiate MVPA from sedentary and other lesser activity and identify sedentary behavior. In addition to activity monitoring, the BioButton device was also able to differentiate upright vs. recumbent positioning, which may be relevant to children with mobility and activity limitations. Physical activity represents an important outcome domain for children who have experienced critical illness, and the ability to measure activity in free-living conditions is an important complementary evaluation to survey data, which is currently the most common method of assessment of physical outcomes after critical illness. The BioButton device can serve as an alternative option to measure physical recovery longitudinally and could provide a method to identify patients who require further interventions after critical illness.

Core outcome sets developed for critically ill children and adults highlight the importance of physical recovery (7–9, 32, 33). In core outcome sets for adults, surveys are most commonly used for physical activity assessment, but there are objective measures also including the 6 min walk test, short physical performance battery; and 30 s sit to stand test (7, 34–36). In core outcome sets for children who have survived an episode of critical illness, physical activity measurements are limited to survey-based tools (8, 37). Commonly used measures to evaluate physical functioning are the Functional Independence Measure including the pediatric version, WeeFIM, the Functional Status Scale (FSS) score, the PedsQL Physical Health Summary Score, which is a HRQL measure, and the Pediatric Evaluation of Disability Inventory Computer Adaptive Test (PEDICat) (2, 4, 38–41). These measures can reflect change in function over time but are limited by subjectivity. Researchers evaluating physical health recovery after critical illness could add objective physical health data using mobile monitoring devices. As demonstrated by the survey data collected at the end of the wear period of our study, the BioButton may be an acceptable device for this use due to the simplicity of placement and removal as well as the discrete design.

The remote set-up and monitoring capabilities provided by the BioButton and ActiGraph accelerometer may reduce disparities in measuring physical health outcomes by eliminating the need to return to a hospital or clinic for direct evaluation. Removing this barrier may increase participation of socially vulnerable populations who are underrepresented in research but overrepresented in PICU patients (42, 43). Adoption of these methods in critical care research as well as other domains of pediatric research may provide more robust trial outcomes and improve research access for underrepresented populations. Additionally, BioButton users can access their data through a smartphone and data transmission through cellular networks allows for data collection even if the device is not returned. The single-use and relatively low cost of the BioButton, currently one-fifth the cost of the ActiGraph accelerometer, are also attractive features. Additionally, there may be opportunities to pair BioButton monitoring with electronic ecological momentary assessments to understand the social and environmental contexts associated with more sedentary or more active periods (44–47). However, there are important limitations of this technology to quantify physical activity outcomes. The lack of pre-illness baseline measurements poses a challenge to evaluating the impact of critical illness on objectively measured physical outcomes. Additionally, use of the BioButton device requires connection to the downloadable BioMobile™ application by a smart phone or connection to a separate BioHub™ device for wireless data transmission. Notably, BioButton users can access their information through the BioMobile™ application, which could influence their overall physical activity. This would be important to consider if used as a trial outcome. Lastly, the BioButton smartphone application, user guides, and related resources are currently only available in English.

In addition to measuring physical activity, the BioButton device's ability to characterize body position may provide a valuable measure of physical outcomes in children with mobility restrictions. A recent epidemiologic study of critically ill children identified that nearly 60% of children have a pre-existing comorbidity including one in four with technology dependence and one in five with a neuromuscular comorbidity (1). These patients frequently have mobility limitations, but their physical functioning outcomes could be readily quantified by positioning including time in the upright position. The BioButton device's ability to differentiate position offers a unique opportunity to quantify this physical outcome for children with limited mobility.

This study has important limitations. Although we attempted to capture activity and positioning data at the minute-level using direct observation, start and stop times of activity may not have been exact, particularly due to the need for water breaks and brief rests during significant heat on some study days. Due to study location, we were unable to use a gold-standard method to evaluate exercise intensity (e.g., indirect calorimetry). Comparison with the ActiGraph accelerometer demonstrated acceptable specificity to discriminate accelerometer-identified MVPA, even though sensitivity was lower in the older age groups. This may be due to the wrist-worn location for the ActiGraph accelerometer in older participants relative to the similarity of truncal locations between the ActiGraph accelerometer in younger patients (hip) and the BioButton (chest). Discrimination of accelerometer-identified sedentary behavior based on BioButton thresholds was good. Our study population was limited to healthy, typically developing children, which may limit generalizability to children with physical or cognitive impairments. Lastly, the variability in exertion by study participants who completed the same type of activity may also have affected our study's internal validity when comparing measured to documented activity. We attempted to mitigate this with direct one-on-one interaction with the children to encourage participation at the appropriate activity level and note when the activity level was not reached but some participants struggled to maintain specific activity levels for prolonged periods of time.

The BioIntelliSense BioButton® device is a promising new technology that may serve as an accurate, objective, and feasible method to measure physical outcomes in children. This device or devices with similar characteristics may serve as a method to measure objective, patient-centered outcomes for future clinical trials to complement patient-reported outcome measures. Future studies are needed to evaluate tolerance and accuracy of prolonged monitoring periods.

## Data Availability

The raw data supporting the conclusions of this article will be made available by the authors with appropriate approvals, without undue reservation.
