# Supplementary material for: Validation of the BioIntelliSense BioButton® device for physical activity monitoring in children and future application as a physical health outcome for critically Ill children
Source: Front Pediatr. 2025 Apr 15;13:1544404. doi: 10.3389/fped.2025.1544404 (PMC12037551; doi:10.3389/fped.2025.1544404)
Supplement: Supplementary file 1 [file Table1.docx]

**Supplemental Material for** Validation of the BioIntelliSense BioButton® Device for Physical Activity Monitoring in Children and Future Application as a Physical Health Outcome for Critically Ill Children

Lexi Petruccelli, BA, Kristen R. Miller, MS, Rachel Greer, BA, Heidi Sauceda, EdS, MS, R. Scott Watson, MD, MPH, Peter M. Mourani, MD, Aline B. Maddux, MD, MSCS

Table of Contents

eTable 1. Activities conducted during monitoring period.

eTable 2. Satisfaction Survey Questions

eTable 3. Activity Count Thresholds

eTable 4. BioButton Identification of Body Position

eTable 5. Discrimination for Identification of Body Position

eTable 6. Body Position Agreement Overall

**eTable 1. Activities conducted during monitoring period**

| **Activity Level** | **Duration** | **Activity Examples** |
| --- | --- | --- |
| Sedentary^a^ | 10 minutes | Laying down  Writing or coloring |
| Light | 7 minutes | Slow Walk |
| Moderate | 10 minutes | Passing a soccer ball  Throwing a frisbee |
| Vigorous | 7 minutes | Active playing on a playground (e.g., trampoline)  Running |
| Sedentary^a^ | 5 minutes | Sitting upright |
| ^a^During sedentary times, positions (supine, prone, side-laying) were directed and recorded. | | |

**eTable 2. Satisfaction Survey Questions**

| **Question** | **Answer Options** |
| --- | --- |
| The BioButton was comfortable for my child to wear while active. | Strongly agree, Agree, Disagree, Strongly Disagree |
| The BioButton was comfortable for my child to wear while laying down on their back or side. | Strongly agree, Agree, Disagree, Strongly Disagree |
| It was easy to place the BioButton on my child. | Strongly agree, Agree, Disagree, Strongly Disagree |
| It was easy to remove the BioButton. | Strongly agree, Agree, Disagree, Strongly Disagree |
| I would agree to a research study that involved my child wearing the BioButton for 1 week at 3 separate time points. | Strongly agree, Agree, Disagree, Strongly Disagree |
| Please add any other comments you or your child have about the BioButton. | *Free text* |
| The ActiGraph accelerometer was comfortable for my child to wear while active. | Strongly agree, Agree, Disagree, Strongly Disagree |
| The ActiGraph accelerometer was comfortable for my child to wear while laying down on their back or side. | Strongly agree, Agree, Disagree, Strongly Disagree |
| It was easy to place the ActiGraph accelerometer on my child. | Strongly agree, Agree, Disagree, Strongly Disagree |
| It was easy to remove the ActiGraph accelerometer. | Strongly agree, Agree, Disagree, Strongly Disagree |
| I would agree to a research study that involved my child wearing the ActiGraph accelerometer for 1 week at 3 separate time points. | Strongly agree, Agree, Disagree, Strongly Disagree |
| Please add any other comments you or your child have about the ActiGraph accelerometer. | *Free text* |

**eTable 3. Activity Count Thresholds**

| **Positive Category** | **Negative Category** | **Optimal Threshold** |
| --- | --- | --- |
| Light | Sedentary | 1303178 |
| Moderate | Light | 3585344 |
| Vigorous | Moderate | 7241588 |

**eTable 4. BioButton Identification of Body Position**

| **Body Position Data Agreement, n (%)** | **Total Minutes** | **Active Minutes** | **Sedentary Minutes** |
| --- | --- | --- | --- |
| Total Body Position Minutes | 1432 | 785 | 647 |
| Agreed | 1125 (78.6) | 721 (91.8) | 404 (62.4) |
| Did not agree | 307 (21.4) | 64 (8.2) | 243 (37.6) |
| Minutes during Upright Position | 947 | 784 | 163 |
| Agreed | 861 (90.9) | 721 (92.0) | 140 (85.9) |
| Did not agree | 86 (9.1) | 63 (8.0) | 23 (14.1) |

**eTable 5. Discrimination for Identification of Body Position**

| **Position** | **Sensitivity**  **(95% Confidence Interval)** | **Specificity**  **(95% Confidence Interval)** |
| --- | --- | --- |
| Upright | 0.91 (0.88, 0.94) | 0.76 (0.69, 0.83) |
| Recumbent | 0.76 (0.69, 0.83) | 0.91 (0.88, 0.94) |

**eTable 6. Body Position Agreement Overall**

| **Minutes of body position measured by the BioButton, n (%)** | **Activity Log Body Position** | | | |
| --- | --- | --- | --- | --- |
|  | **Lateral (n=110)** | **Prone**  **(n=140)** | **Supine**  **(n=235)** | **Upright**  **(n=947)** |
| Lateral | 63 (57.3) | 25 (17.9) | 13 (5.5) | 22 (2.3) |
| Prone | 6 (5.5) | 103 (73.6) | 13 (5.5) | 52 (5.5) |
| Supine | 40 (36.4) | 6 (4.3) | 98 (41.7) | 12 (1.3) |
| Upright | 1 (0.9) | 6 (4.3) | 111 (47.2) | 861 (90.9) |
